# Less fear, more diversity

**DOI:** 10.1371/journal.pbio.2002079

**Published:** 2017-04-17

**Authors:** Gregory J. Quirk

**Affiliations:** Departments of Psychiatry and Anatomy & Neurobiology, University of Puerto Rico School of Medicine, San Juan, Puerto Rico

## Abstract

Fear is an instinctual response that’s adaptive and critical for survival when it is short-lived but can lead to anxiety disorders when chronic. Studying how the brain controls our fears helps us understand the mechanisms required to recover from traumatic experiences and what goes wrong when we don’t. Research in rodents has identified neural circuits and molecular mechanisms regulating fear expression. Rodent work has been amenable to translation to humans and has led to improvements in clinical therapies for anxiety disorders. The societal benefit of this type of research is magnified when performed in minority-serving institutions, offering high-caliber training opportunities to increase ethnic diversity in science.

## Less fear

Who likes to be afraid? Fans of horror movies and roller coasters revel in the exhilaration of a recreational fright, but exposure to real danger can be truly terrifying, eliciting a range of powerful responses including fearful thoughts and physiological changes. Although the fear triggered by movies and roller coasters is powerful, its playful nature leaves no emotional scars, unlike the fears associated with traumatic encounters, demonstrating that fear associations are heavily regulated by our thoughts. Appropriate regulation of fear keeps us calm while at the same time enabling us to detect and avoid threats in the environment. Abnormal fear regulation leads to excessive anxiety and fears associated with phobias and posttraumatic stress disorder (PTSD). A better understanding of the brain mechanisms that regulate learned fear will help us find ways to alleviate these conditions.

Traumatic events can form lifelong associations of the event with specific cues, which can later trigger fear responses. The amygdala—a pair of symmetrical clusters of neurons located deep within the brain—is the key site of storage and expression of these associations and is heavily controlled by inputs from the medial prefrontal cortex (mPFC), a region of the brain involved in decision making, executive control, and reward-guided behaviors. Using a well-established rodent model of fear learning, in which rats learn to freeze in response to a tone that predicts a shock, we showed years ago that neurons in a specific part of the mPFC are activated when the tone is repeatedly presented without the shock, a process that can lead to fear extinction [1]. Extinction of fear is the ability to learn that a previously dangerous stimulus no longer carries a risk and functions as a form of healing after trauma. Over the past ten years, a steadily increasing number of rodent studies employing recording, stimulation, pharmacological, and optogenetic techniques have characterized how subdivisions of the mPFC regulate fearful memories via projections to the amygdala as well as to other areas such as the thalamus and brainstem [2,3,4,5]

Fortunately, circuits regulating fear are fundamentally similar across species [6], and predictions from rodents can be tested in humans using functional magnetic resonance imaging (fMRI)—a noninvasive technique that measures brain activity by monitoring changes associated with blood flow. This approach has revealed that the amygdala works in conjunction with prefrontal and other cortical areas to regulate laboratory-generated fear responses [7]. Furthermore, connectivity between these areas is altered in individuals with anxiety disorders. Activity and connectivity measures from brain imaging could serve as biomarkers predicting disease onset or a patient’s response to a specific type of therapy [8].

Extinction of fear is the foundation of behavioral therapies for PTSD, in which patients are repeatedly exposed to trauma-related stimuli in a safe environment. Indications that extinction could be facilitated came years ago, when rodent studies showed that extinction learning depends on the *N*-methyl-d-aspartate (NMDA) type of glutamate receptors, a postsynaptic receptor that mediates excitatory communication between neurons [9]. Facilitating NMDA receptors with a partial agonist (D-cycloserine) accelerates fear extinction in rats and was later shown to augment patients’ response to exposure therapy [10]. Other drugs such as estradiol, a female sex hormone produced by the ovaries, facilitates extinction in rodents and can be explored as an adjunct to exposure therapy [11]. Surprisingly, the timing of extinction delivery seems to be very important, as the effectiveness of extinction varies with estrous and sleep cycles. Furthermore, initiating extinction within a brief time window following reactivation of a traumatic memory appears to erase that fear memory [12], which is the holy grail for anxiety treatment. These results show that basic insights into brain function from rodent studies of fear regulation can improve our understanding of the human condition and help reduce human suffering.

## More diversity

Important for many scientists is the larger question of what brings meaning to their daily work. For me, meaning flows from the training and mentoring aspects of science. Where do I do my research, with whom, for whom? My choice of Puerto Rico grew out of this. After finishing graduate training in Brooklyn, New York, in the early 90s, I became aware of two noticeably unfair aspects of scientific practice in the West: little participation by ethnic minorities in the United States, and relatively little participation by developing countries. I decided to address both issues in my own career. Armed with a Fulbright grant, I went to Tegucigalpa, Honduras, to start a neurophysiology laboratory at the National Medical School [13]. My one-year stint in Honduras opened my eyes to the opportunities and challenges of doing research in a developing country. Following postdoctoral training at New York University, I realized that I needed to spend more than a year in Latin America in order to make a significant contribution.

Establishing my lab in Puerto Rico was relevant to both unfairness issues in science. In the United States, the percentage of Latino researchers and Latino federal grant recipients is extremely low despite the sharp increase in Latinos in the general US population [14]. Twenty years after arriving, I’ve trained over 50 highly motivated undergrads, graduate students, and postdoctoral fellows from Puerto Rico and other Latin countries. Most of these young people have continued their training in the US, and some have obtained faculty or administrative positions in the US, Puerto Rico, and other parts of Latin America. While Puerto Rico is legally part of the US, it is very much its own country with its own cultural heritage. Unfortunately, it suffers from the “brain drain” afflicting many developing countries, losing many of its best and brightest young people to more developed countries. One solution to this problem is to establish first-class training sites in-country so that young people interested in neuroscience are not forced to leave their languages, families, and cultures so early in their careers. In Puerto Rico, this has meant obtaining competitive grants and fellowships, exposing trainees to international conferences and colleagues, and publishing their work in high-impact journals. All of this enables Puerto Rico to more equitably compete for scientific resources and research opportunities and take its rightful “place at the table” in neuroscience.

While in San Juan, I have been inspired by colleagues in other parts of the globe who founded successful neuroscience laboratories in Bangalore, Mexico City, Natal, Manila, Montevideo, Maracaibo, Santiago, and other places at a competitive disadvantage compared to the West. Some of my colleagues at prestigious institutions expressed doubts and concerns about the feasibility of this project at its early stages, but as the psychologist Carl Jung once said, “The least of things with a meaning is worth more in life than the greatest of things without it” [15]. Perhaps meaning has been my most important scientific discovery to date.

## Abbreviations

fMRI: functional magnetic resonance imaging
mPFC: medial prefrontal cortex
NMDA: *N*-methyl-d-aspartate
PTSD: posttraumatic stress disorder
